# Stress Response and Perinatal Reprogramming: Unraveling (Mal)adaptive Strategies

**DOI:** 10.1155/2016/6752193

**Published:** 2016-03-16

**Authors:** Laura Musazzi, Jordan Marrocco

**Affiliations:** ^1^Laboratorio di Neuropsicofarmacologia e Neurogenomica Funzionale, Dipartimento di Scienze Farmacologiche e Biomolecolari and CEND, Università degli Studi di Milano, 20133 Milano, Italy; ^2^Harold and Margaret Milliken Hatch Laboratory of Neuroendocrinology, The Rockefeller University, New York, NY 10065, USA

## Abstract

Environmental stressors induce coping strategies in the majority of individuals. The stress response, involving the activation of the hypothalamic-pituitary-adrenocortical axis and the consequent release of corticosteroid hormones, is indeed aimed at promoting metabolic, functional, and behavioral adaptations. However, behavioral stress is also associated with fast and long-lasting neurochemical, structural, and behavioral changes, leading to long-term remodeling of glutamate transmission, and increased susceptibility to neuropsychiatric disorders. Of note, early-life events, both* in utero* and during the early postnatal life, trigger reprogramming of the stress response, which is often associated with loss of stress resilience and ensuing neurobehavioral (mal)adaptations. Indeed, adverse experiences in early life are known to induce long-term stress-related neuropsychiatric disorders in vulnerable individuals. Here, we discuss recent findings about stress remodeling of excitatory neurotransmission and brain morphology in animal models of behavioral stress. These changes are likely driven by epigenetic factors that lie at the core of the stress-response reprogramming in individuals with a history of perinatal stress. We propose that reprogramming mechanisms may underlie the reorganization of excitatory neurotransmission in the short- and long-term response to stressful stimuli.

## 1. Introduction

Life experiences often produce uncertainty or threat and trigger a physiological response, the so-called “stress response,” aimed at promoting adaptation and improving survival [1]. The stress response, including fast and transient activation of the autonomic nervous system and of the hypothalamic-pituitary-adrenocortical (HPA) axis, implies release of catecholamines and corticosteroids (mainly cortisol in humans and corticosterone in rodents). Corticosteroids exert their function through the activation of the high-affinity mineralocorticoid receptor (MR) and the low-affinity glucocorticoid receptor (GR), which are widely expressed both at peripheral level and in the brain. Corticosteroids, together with regulating metabolism, food intake, and the immune system, modulate brain function, neuronal transmission, and plasticity, especially in corticolimbic areas [2, 3].

In the high majority of individuals, the stress response is able to activate coping strategies to adverse environmental changes, promoting stress resilience. However, in vulnerable subjects, the stress response may become dysregulated and induce maladaptive changes, which in turn underlie increased susceptibility to stress-related neuropsychiatric diseases [4–6]. Daskalakis and collaborators [7] proposed the 3-hit concept (hit 1: genetic predisposition, hit 2: early-life environment, and hit 3: later-life environment) to explain why some individuals can cope with adverse events and remain resilient while others are vulnerable and succumb to stress-related disorders. This concept readapts the cumulative hypothesis of stress [8], which indicates that accumulating failures to cope with stressors lead to dramatic consequences on the individuals, consistent with increased vulnerability to psychiatric disorders [1]. Of note, the 3 hits also endorse the mismatch hypothesis of psychiatric disorders, suggesting that early-life adversities can prepare the individuals to cope with future life similar challenges; conversely, the coping strategies are compromised when the later-life events exhibit mismatch with the early-life environment [9, 10].

In this context, during the last decades, there have been a growing number of studies on short- and long-term consequences of early-life stress (including manipulations during the prenatal period and/or the early phase of postnatal development), suggesting an increased interest in the impact of early-life adversities on stress response and susceptibility to neuropsychiatric disorders in the adulthood [11–24].

In the first sections of the present review, we will summarize functional, morphological, and epigenetic changes in adults induced by stress exposure during adulthood, or during early life. Finally, in order to recapitulate the interplay between life adversities at early stages and in the adulthood, we will introduce the concept of “reprogramming,” a process whereby a stimulus or insult, during a sensitive period of development, has lasting and/or lifelong significance, inducing readaptation of the stress response.

## 2. Acute and Chronic Stress at Synapses: Corticosterone-Dependent Effects of the Stress Response

A growing body of literature has analyzed the multifaceted effects of different stress protocols and of corticosteroids (mainly corticosterone) on neurotransmission, neuronal plasticity, and behavior (see below). MR and GR are nuclear receptors, acting as transcription factors, ultimately leading to regulation of gene expression. However, more recently, compelling evidence has reported fast effects of corticosteroids on neuronal excitability, in line with early nongenomic mechanisms that are likely dependent on membrane-located receptors (for recent reviews, see [25–27]).

In the next sections, we will review the most recent findings on fast and delayed effects of acute and repeated stress, mediated by genomic and nongenomic action of corticosteroids in the brain.

### 2.1. Corticosterone-Dependent Effects of Stress on Excitatory Neurotransmission

A number of studies have been performed to unravel the time-dependent and brain area-specific effects of stress on neuronal excitability and cognitive processes (for recent reviews, see [2, 26–28]). The changes in neuronal excitability and synaptic plasticity induced by stress are the result of an imbalance of excitatory (glutamatergic) and inhibitory (GABAergic) transmission, leading to long-lasting (mal)adaptive functional modifications [28–34]. Although both glutamate and GABA transmission are critically associated with stress-induced alteration of neuronal excitability [32, 34], the present review will focus on the modulation of glutamate release and transmission induced by stress and glucocorticoids.

Genomic and nongenomic effects of acute stress were characterized in both the hippocampus and the prefrontal cortex. Acute stress was consistently reported to rapidly enhance the frequency of miniature excitatory currents (mEPSCs) at hippocampal synapses, thus suggesting increased probability of glutamate release, through nongenomic action of corticosterone, and activation of membrane-located pre- and postsynaptic MR [29, 35–37]. On the other hand, slower genomic effects of acute stress in the hippocampus are mainly mediated by GR, which prevents synaptic *α*-amino-3-hydroxy-5-methyl-4-isoxazolepropionic acid (AMPA) receptor potentiation and enhances voltage-dependent calcium currents and mEPSCs amplitude. This leads to steady depolarization and attenuation of firing activity [29, 38, 39] and impaired long-term potentiation (LTP) [30, 40].

Partially different effects of acute stress were reported in the prefrontal cortex, where nongenomic mechanisms, despite being involved in the priming of excitatory synapses, are not sufficient to induce changes in glutamate release and transmission [41–43]. Indeed, it was found that corticosterone rise induced by acute footshock stress increases the size of the readily releasable pool of glutamatergic vesicles in the prefrontal cortex, through completely nongenomic mechanisms involving both MR and GR located at synaptic sites. However, in the same brain area, slower genomic mechanisms are required to enhance presynaptic glutamate release and mEPSCs amplitude [43–45]. In line with these observations, different acute stress protocols, as well as acute corticosterone* in vivo* and* in vitro* treatments, were shown to induce delayed and long-lasting increase of excitatory transmission in prefrontal cortex pyramidal neurons [44–46].

In recent studies, fast and slow effects of acute stress were also analyzed in the amygdala [47]. In basolateral amygdala, a brain area responsible for the control of emotion and fear memory, different and partially opposite effects of stress compared to hippocampus and prefrontal cortex were reported. Indeed, the early nongenomic increase of mEPSCs frequency induced by corticosterone is accompanied by a fast decrease in synaptic potentiation (LTP), while genomic and delayed effects include enhancement of neuronal excitability and synaptic plasticity.

Long-term adaptive changes induced by repeated stress exposure include impairments in neuronal transmission and synaptic activity (extensively reviewed in [27, 31, 32, 48]). Indeed, in both hippocampus and prefrontal cortex, chronic stress was associated with defects in the ability to induce or maintain LTP, and increased long-term depression (LTD), together with enhancing basal transmission, while opposite changes were measured in basolateral amygdala. The changes in neuronal activity induced by chronic stress in brain areas involved in the negative feedback of the HPA axis are consistent with impairment and dysregulation of the stress response [31]. It has been suggested that functional connectivity between amygdala and ventral hippocampus plays a key role in stress-induced changes in synaptic plasticity (for a recent review, see [49]). In particular, Ghosh and coworkers showed that, in animals subjected to repeated restraint stress, the directional connection from the amygdala to the hippocampus is gradually and persistently potentiated [50]. The authors suggest that this mechanism could be involved in the long-term emotional and cognitive impairments induced by chronic stress.

### 2.2. Morphological and Cytoarchitectural Changes Induced by Stress

Stress and corticosterone cause structural alterations, including dendritic remodeling and changes in spine density, mostly in brain areas implicated in the regulation of the emotional state (for recent reviews, see [27, 51, 52]).

Chronic stress was reported to reduce dendritic arborization and synaptic contacts both in hippocampus and in prefrontal cortex, whereas in basolateral amygdala, both chronic and acute exposure to stressors significantly increased dendritic complexity (for recent reviews, see [27, 48–53]). These long-lasting structural changes occur together with increased anxious- and depressive-like behaviors, strongly suggesting that dendritic atrophy induced by chronic stress may induce severe behavioral deficits [48, 53, 54].

Recently, a few studies also analyzed morphological alterations induced by acute stress and corticosterone. Acute corticosterone treatment of rats was shown to induce delayed and time-dependent opposite changes of dendrite morphology in medial prefrontal cortex pyramidal neurons, compared to basolateral amygdala spiny neurons [55]. Similarly, 5 hours of multimodal combined physical/psychological stress was demonstrated to induce a corticotropin-releasing hormone-dependent reduction of spine density in hippocampal area CA3 [54]. In a more recent study, one single session of acute footshock stress was shown to reduce the apical dendritic length of pyramidal neurons in medial prefrontal cortex layers II-III [56]. Intriguingly, this effect was measured as early as one day after stress and lasted for up to 14 days. Furthermore, the effect was partly prevented by chronic treatment with antidepressants before the stress session.

On the other hand, in line with the rapid enhancement of excitatory transmission induced by stress and improvement of working memory performance [44, 45], both acute footshock and acute restraint stress were shown to remarkably increase the number of excitatory axoshaft and axospinous synapses in the medial prefrontal cortex of rats [57] and to induce sprouting of new spines one day after stress [56]. Accordingly, Liston and Gan [58] have shown that acute treatment with corticosterone promotes a dose-dependent increase of spine formation in medial prefrontal cortex pyramidal neurons. Furthermore, acute corticosterone induces rapid GR-dependent spinogenesis in hippocampal slices [59]. These findings suggest that the increased number of synapses induced by acute stress is likely a corticosterone-dependent effect.

### 2.3. Epigenetic Changes Induced by Stress

The term “epigenetics” refers to mechanisms modulating gene expression independently of changes in nucleotide sequence and includes alterations of DNA methylation, posttranslational modification of histone proteins, and regulation by small noncoding RNAs (essentially, microRNAs, miR) [60, 61]. Compelling evidence showed that behavioral stress induces epigenetic changes in selected brain areas, leading to regulation of gene expression and neuronal function [61–63].

A few studies have assessed changes in DNA methylation (a modification associated with gene silencing) in stress animal models. Chronic social stress in mice induced persistent demethylation at the corticotrophin-releasing factor promoter in the paraventricular nucleus of the hypothalamus, suggesting hyperactivation of the HPA axis [64]. Moreover, in a recent study, changes in the global DNA methylation profile were measured after acute restraint stress in the hippocampus, cerebral cortex, and periaqueductal gray matter, while these alterations were prevented by physical exercise [65].

A growing body of literature reported posttranslational modification of histone proteins after exposure to acute and chronic stress protocols. A genome-wide chromatin immunoprecipitation study reported changes in histone H3 lysine 9 dimethylation levels (inducing repression of gene expression) in the nucleus accumbens of mice susceptible to chronic social defeat stress, and not in resilient animals [66, 67]; importantly, chronic antidepressants reversed these modifications [68]. A recent study also showed that both chronic social defeat in mice and depression in humans reduced the expression of the RAS-related C3 botulinum toxin substrate 1 (Rac1) gene in the nucleus accumbens, through a mechanism involving increased histone H3 lysine 9 dimethylation [69]. On the other hand, permissive histone H3 acetylation is transiently reduced and then persistently increased in the nucleus accumbens of susceptible, but not of resilient, animals subjected to chronic social stress [66]. In the same paper, similar results were obtained in postmortem studies, reporting increased levels of histone H3 acetylation in the nucleus accumbens of depressed patients. However, since local infusion of histone deacetylase inhibitors showed an antidepressant-like effect, the authors hypothesized that the increase in H3 acetylation measured in susceptible animals might mediate long-lasting positive neuronal adaptations to chronic stress.

In the hippocampus, selected and time-dependent changes in histone H3 methylation at lysines 4, 9, and 27 (resp., associated with increased transcription, heterochromatin formation, and transcriptional repression) were demonstrated after acute and repeated restraint stress in rats [70]. A further study from the same group showed that, soon after one single session of restraint stress, repressive histone H3 lysine 9 trimethylation is selectively increased in the hippocampus, especially at transposable element loci [71]. Individual variations of histone H3 acetylation levels were also reported in the hippocampus of rats subjected to repeated social defeat stress [72, 73]. Moreover, it was shown that the acquisition of behavioral immobility response induced by acute forced swim stress was dependent on increased histone H3 phosphoacetylation in the hippocampus and GR-induced activation of the NMDA/extracellular signal-regulated kinases (ERK)/mitogen- and stress-activated kinases (MSK) 1/2 pathway [74].

Repeated social defeat stress was also found to increase histone H3 acetylation in the infralimbic (and not prelimbic) prefrontal cortex [73, 75]. A recent study on postmortem PFC from patients with mood disorders reported increased levels of the presynaptic protein synapsin 2, together with increased histone H3 lysine 4 trimethylation at its promoter, suggesting epigenetic regulation of synapsin 2 gene expression [76].

Intriguingly, a number of studies reported stress-induced epigenetic regulation of the brain-derived neurotrophic factor (BDNF), a neurotrophin with key roles in neuroplasticity and synaptic function, as well as in the pathophysiology of neuropsychiatric disorders [77, 78]. The expression of BDNF is mediated by the transcription of different mRNAs, driven by dedicated promoters and derived by the splicing of one of multiple 5′ noncoding exons (at least eight in rodents) with the 3′ coding exon [79].

Social defeat stress induced long-lasting downregulation of BDNF transcripts containing exons IV and VI, by increasing dimethylation of histone H3 at specific exon promoters in the mouse hippocampus, and chronic imipramine reversed this downregulation increasing histone acetylation at the same promoters [80]. Similarly, the reduction of total BDNF transcript and mRNAs containing exons I and IV expression, induced by single immobilization stress in the rat hippocampus, was shown to be accompanied by a significant decrease in histone H3 acetylation at respective promoters [78]. In a more recent study, physical exercise was found to counteract the downregulation of selected BDNF transcripts induced by acute restraint stress and to increase the levels of histone H3 acetylation at related promoters [81].

MicroRNAs are small noncoding RNAs regulating gene expression, generally repressing the expression of target mRNAs [82]. In recent years, research studies have been conducted on the involvement of microRNAs in the stress response and onset of neuropsychiatric disorders [83]. It was shown that both acute restraint stress and chronic social defeat in mice markedly upregulated miR-34 levels in amygdala and that miR-34 overexpression in the central amygdala exerted anxiolytic effect [84]. In the same study,* in vitro* experiments showed that miR-34 reduced the activation of the corticotropin-releasing hormone receptor 1, suggesting a role of miR-34 in functional regulation of the stress response. In more recent papers from the same research group, miR-135 in serotonergic neurons was found to have a key role in determining stress resiliency and antidepressant efficacy [85], while the increase of amygdalar miR-19b induced by chronic social defeat stress was suggested to be related to behavioral responses to stress, through mechanisms involving the adrenergic receptor *β*-1 [86].

## 3. Perinatal Reprogramming of the Stress Response

The high majority of functional and morphological changes promoted by behavioral stress and corticosteroids were reported in “naïve” young adult animals or mature neuronal cultures. Nevertheless, early-life experiences shape the stress response in adulthood, leading to the reprogramming of coping strategies against environmental challenges and having a strong impact on behavior and susceptibility to neuropsychiatric disorders (see Section 1).

Intriguingly, a few studies on humans aimed at separating the effects of the objective exposure to a stressor and the mother's subjective reaction [87–90]. According to King and Laplante [87], exposure to a natural disaster (Project Ice Storm) occurring during the gestational period allows for a reliable study of the effects of prenatal stress on child health and development [91].

However, the reprogramming effects observed in the offspring likely recapitulate the cumulative experience* in utero* and the quality of the postnatal environment, which is, in turn, mostly associated with the quantity, quality, and reliability of maternal care [92–97]. Thus, we will refer to “perinatal” reprogramming to include events occurring prenatally and/or during the lactation period. Considering that the limitations of retrospective studies constrain the number of epidemiological findings in humans, a large number of data come from evidence in rodents and nonhuman primates [21, 24, 98–104].

### 3.1. Changes in Excitatory Neurotransmission Induced by Perinatal Stress

Overall, the changes in excitatory transmission and neuronal remodeling, induced by both acute and chronic stress (reviewed in Section 2.1), strongly suggest a key role of the glutamate synapse in the adaptive and maladaptive response to stressful stimuli. However, the study of the effects of exposure to perinatal stress on the activity of glutamatergic neurons is still at its infancy.

Morphological studies have shown that prenatal stress is associated with reduction of dendritic arborization and synaptic loss in prefrontal cortex and hippocampus in adult life, suggesting that stress in gestational period might induce long-lasting impairments of glutamate neuron and transmission [105–108].

A number of studies reported changes in the expression of glutamate receptors and transporters, in adult animals subjected to stress during the perinatal life [109–115]. Maternal separation in rats was found to decrease mRNA expression levels of ionotropic glutamate receptors, together with increasing GLutamate ASpartate Transporter (GLAST) levels, selectively in the hippocampus and not in the prefrontal cortex [109]. It was also demonstrated that maternal separation significantly reduced the expression of type 4 metabotropic glutamate receptor in hippocampus, a change reversed by chronic fluoxetine treatment [110]. Similarly, adult male offspring of pregnant dams subjected to restraint stress during pregnancy display impairment of N-methyl-D-aspartate (NMDA) receptor-mediated long-term potentiation, decreased NMDA receptor subunits [111], and reduced expression of group I/II metabotropic glutamate receptors [112] in the hippocampus. In a more recent study, Adrover and collaborators [113] have shown increased mRNA and protein expression levels of the glial glutamate transporter (GLT-1) in the hippocampus and enhanced glutamate uptake and vesicular glutamate transporter 1 (v-Glut-1) protein levels in the prefrontal cortex of prenatally stressed rats.

Overall, the high majority of studies reported that early stress both decreases the expression of glutamate receptors, suggesting reduced transmission efficacy, and increases glutamate transporters, which may imply an increased rate of glutamate metabolism. Of note, others have found higher levels of ionotropic and metabotropic glutamate receptors [114] and increased NMDA receptor activation [115]. Others have shown impairment of long-term potentiation and enhancement of long-term depression in young rats subjected to prenatal stress [116]. These abnormalities were correlated with increased pro-brain-derived neurotrophic factor (pro-BDNF), decreased mature BDNF levels, and no changes in NMDA receptor subunits expression [116]. Although changes in the expression of glutamate receptors and transporters are only rough indicators for predicting glutamate release and transmission, these data strongly suggest that perinatal stress exerts a long-term influence on the glutamate system.

It was recently demonstrated that the increase of anxiety-like behavior induced by prenatal stress in rats is causally associated with a reduction of depolarization-evoked presynaptic glutamate release in the ventral hippocampus [117, 118], a brain region encoding memories related to stress and emotions [119]. Interestingly, this effect is blocked by activation of oxytocin receptor [120] (see below for oxytocin and reprogramming). Although the mechanisms by which prenatal stress may cause long-lasting dampening of glutamate neurotransmission in the ventral hippocampus have been poorly clarified, it was hypothesized that prenatal stress, besides enhancing glutamate metabolism, might induce long-lasting dysfunction in the intrinsic machinery controlling exocytotic glutamate release [117].

### 3.2. Modulation of HPA Axis Reactivity Induced by Perinatal Stress

HPA axis alterations are the characteristic feature of the endophenotypes induced by perinatal stress [21, 121–126].

A pioneering study by Levine showed that maternal separation induced downregulation of the stress response, consistent with weight reduction of adrenal glands [127]. To date, the literature about the long-term effects of perinatal stress on the HPA axis is contradictory, although in many species including mice, rats, guinea pigs, and nonhuman primates, prenatal stress has been shown to increase the overall production of glucocorticoid and/or the feedback regulation [100, 101, 103, 128–130]. For example, peer rearing in monkeys has been shown to exaggerate stress reactivity [122, 123], stereotypies and self-directed behaviors [124], and abnormal brain morphology [125]. Moreover, maternal separated rodents show general upregulation of stress and fear responses [126, 131–133], increased hypothalamic CRF expression, reduced cortical GR expression [134], increased immobility in the forced-swim test [135], and poorer memory performance [134]. Curiously, in rats, 3 hours of daily maternal separation during the first two weeks after birth increases the vulnerability to stress in the adulthood [136, 137], whereas 8 hours of separation decreases the response of the HPA axis [138]. Similarly, prenatally restraint stressed rats display prolonged corticosterone secretion associated with downregulation of GR and MR receptors in the hippocampus [21, 92]. Interestingly, these effects are reversed by prenatal adrenalectomy [93] or postnatal cross-fostering [92].

### 3.3. Epigenetic Reprogramming of the HPA Axis: Regulation of GR Expression

An ever growing number of studies focused on short- and long-term epigenetic changes induced by stress in early life (recently extensively reviewed in [22, 92, 139–141]). The mechanisms involved in the epigenetic reprogramming are highly complex and strongly depend on the gender of the individual, the type of stressor, and its intensity and duration. Here, we will focus on the epigenetic regulation of GR in the offspring induced by prenatal and postnatal maternal stress.

At the epigenetic level, the GR gene is consistently affected by natural variation of maternal care in rodents (measured as licking/grooming, arched-back and blanket nursing, and nest building) [142, 143]. Indeed, low absolute levels of maternal care selectively modify the DNA methylation status of GR promoter in the hippocampus of the offspring, suggesting reduced expression of the receptor as well as increased HPA reactivity. Conversely, offspring receiving high levels of maternal care exhibit lower level of DNA methylation of the GR promoter and increased histone H3 lysine 9 acetylation (a marker of transcriptional activation).

The GR gene expression and promoter methylation have also been examined in humans following early-life trauma, with similar epigenetic outcomes. McGowan and collaborators [144] found decreased levels of hippocampal GR mRNA and increased cytosine methylation of the GR promoter in subjects with a history of childhood abuse. Similarly, childhood maltreatment has been associated with decreased hippocampal GR expression and increased stress responses in adulthood. Again, such effects are mediated by DNA methylation and hydroxymethylation across GR promoter regions [145]. A compelling study in genocide survivors suggested that the increased DNA methylation at the promoter region of the GR was associated with less intrusive memory of the traumatic event and sex-specific reduced PTSD risk [146]. Together, these findings indicate that the epigenetic regulation of GR expression is a key factor in the reprogramming of the HPA axis induced by early stress.

### 3.4. Mother-Offspring Interplay: Role of 11*β*-Hydroxysteroid Dehydrogenases and Oxytocin

The mother/infant interaction is a critical intermediary to study early-life reprogramming. Such interplay is mainly mediated by the placenta, which modulates fetal exposure to maternal factors. As an example, glucocorticoids, despite circulating across the placenta, are significantly lower in the fetus than in the mother. This key tissue-specific barrier control is exerted by the placental 11*β*-hydroxysteroid dehydrogenase, an enzyme that converts cortisol and corticosterone into inactive cortisone and 11-dehydrocorticosterone (11*β*-HSD2), and vice versa (11*β*-HSD1) [147–149]. Recently, it has been shown that 11*β*-HSD2 undergoes epigenetic regulation in the placenta and fetal brain [150–152]. Curiously, Appleton and collaborators [153] have shown that women experiencing adversity during pregnancy display low levels of 11*β*-HSD2 methylation. This accounts for increased levels of placental 11*β*-HSD aimed at protecting the fetus from excessive glucocorticoid exposure. Others have shown that pregnant rats exposed to repeated episodes of restraint stress, a model that recapitulates the main feature of anxiety and depression in the adult offspring, display a reduction of 11*β*-HSD2 activity in the placenta, thus increasing the amount of nonmetabolized corticosterone reaching the fetus [55]. Also, high methylation in the promoter region of placental 11*β*-HSD2 has been associated with low infant birth weight [154]. However, it is unclear whether the modifications of the 11*β*-HSD2 are exclusively disruptive and/or directly associated with pathological endophenotypes in late life. For example, the downregulation of 11*β*-HSD2 may provide the fetus with a reliable signal about the maternal stressful environment, thereby predicting the milieu it is likely to cope with after birth.

The maternal HPA axis itself also plays a pivotal role in the mother-offspring interplay. The HPA axis is normally attenuated from midpregnancy to the end of lactation [155–157]; such attenuation is generally associated with maternal behavioral changes including reduced anxiety [158, 159], enhanced maternal behavior [160], and increased aggressiveness [161, 162]. Of note, the central oxytocinergic system exerts this inhibitory effect on the maternal HPA axis [160, 163–167]. Oxytocin is a neurohypophysial peptide, which plays a key role in parturition, lactation, mother/infant interaction, and social behavior [168]. Interestingly, intracerebroventricular administration of oxytocin stimulates maternal behavior in ovariectomized virgin rats [160]. Moreover, enhanced maternal care increases the expression of oxytocin receptor in the central nucleus of the amygdala [169]. Remarkably, in subjects with a history of early-life stress, the inhibitory effect of oxytocin on the HPA axis is diminished or even reversed [120, 170]. Interestingly, impaired social behavior and increased anxiety have been associated with an altered number of oxytocin neurons in the paraventricular and supraoptic nuclei [171] and increased expression of oxytocin receptor in the hippocampus and amygdala [120] of adult prenatally stressed rats. Moreover, it has been shown that the activation of presynaptic oxytocin receptor during the adulthood could both correct the abnormal glucocorticoid feedback of the HPA axis and normalize the expression of GR and MR in the hippocampus in prenatally restraint stressed rats [120]. In humans, it has been shown that intranasal administration of oxytocin dampens the enhanced stress-induced functional connectivity between the amygdala and the hippocampus in subject with a history of early-life stress [172]. Finally, variations in maternal care have been associated with DNA methylation of oxytocin receptor in blood cells both in rodents [173] and in humans [174].

Together, the evidence reviewed in this section emphasizes the molecular and neuroendocrine mechanisms that underlie the critical role of mother-infant interaction in the reprogramming of stress response and vulnerability to neuropsychiatric disorders.

## 4. Conclusion

The “cumulative stress hypothesis” of neuropsychiatric disorders states that repeated exposure to stressful events is the main environmental factor for pathological onset [1, 8, 32]. Thus, it is conceivable that repeated adverse events, especially when added to perinatal stress, exacerbate psychopathological conditions. On the other hand, according to the “match/mismatch hypothesis,” early-life stress might also be somehow protective against stressors in late life, leading to higher achievement of adaptation and survival [7, 9, 175]. Yet, these hypotheses are strictly related and interconnected. Indeed, the deleterious effects of stress rely not only on* when* or* how often* stress occurs, but especially on* how intense* the stress is and* how much* it impacts an individual, depending on one's genetic background [176, 177]. In this context, repeated subjective mild stressors may act improving adaptation to environmental challenges [3, 178], while a single overwhelming adverse event may precipitate neuropsychiatric diseases, as in the case of posttraumatic stress disorder [179, 180].

It is also important to notice that whereas the physiological stress response activates adequate coping strategies, leading to stress resiliency and adaptation in the high majority of subjects, vulnerability toward stress is dependent on individual behavioral, physiological, and genetic factors [4–6]. Thus, individual reprogramming of the stress response induced by early-life stress could be both adaptive and maladaptive. In line with this hypothesis, Macrì and collaborators [181] have suggested that mild neonatal changes may reduce the HPA axis reactivity, leading to resilience, whereas severe neonatal challenges would increase the adult HPA axis reactivity, with the ensuing increased vulnerability to stress-related disorders. Intriguingly, it was recently reported that early-life trauma in humans can also promote early maturation of amygdala-prefrontal cortex connectivity, in line with enhanced emotion regulation and reduced anxious behavior [182].

**Figure 1:**
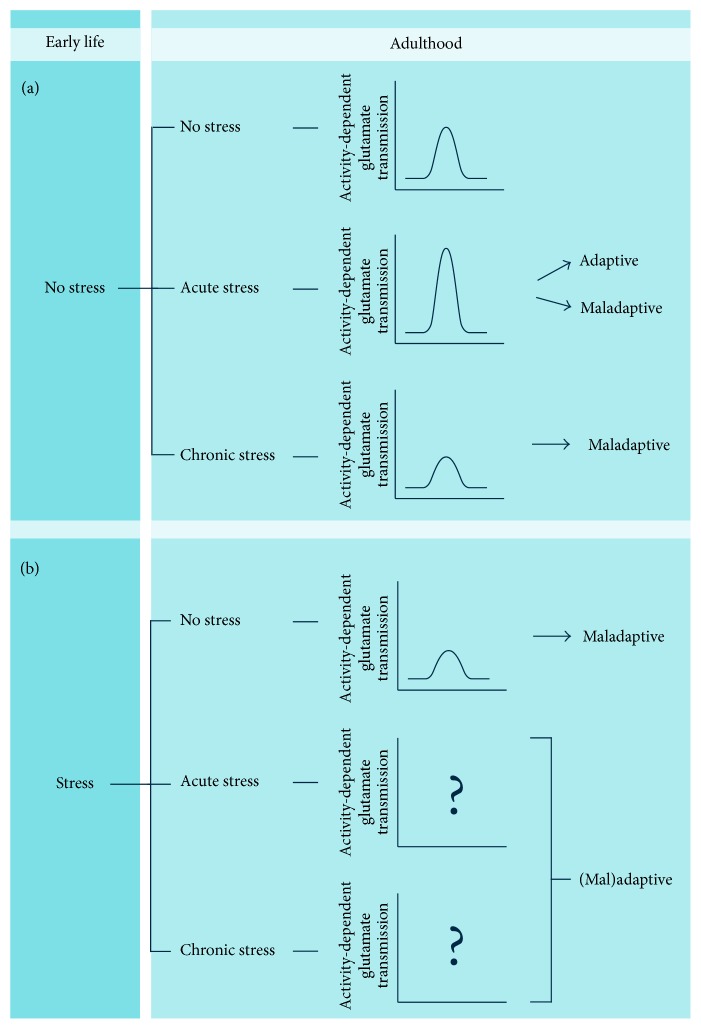
Influence of early-life stress on neuronal excitatory neurotransmission in corticolimbic areas. (a) In subjects with no history of perinatal adverse challenges, acute stress induces an increase in stimulation-evoked glutamate release. This response can be both adaptive and maladaptive. On the other hand, exposure to repeated episodes of stress (chronic stress) induces hypofunction of the glutamatergic synapse with reduced evoked glutamate release, associated with increased vulnerability to stress-related neuropsychiatric disorders. (b) Perinatal stress induces hypofunction of the glutamatergic synapse in adult life, with ensuing reduction in evoked glutamate release. The effects of the association between early- and late-life stress are largely unknown. See text for details.

Integration of perinatal and late-life experiences may induce long-lasting consequences on neuronal excitatory transmission and morphology, especially in corticolimbic areas (Figure 1). If acute stress in adult life was consistently shown to increase glutamate transmission and release, at least in the hippocampus and prefrontal cortex [2, 26–28], chronic stress has the opposite effect, inducing impairments in neuronal transmission and synaptic activity [31] (Figure 1(a)). In turn, the effects of chronic stress are generally associated with behavioral deficits and depressive/anxious-like behavior [32, 61, 183], while the response to acute stress can be both adaptive, with improved behavioral and cognitive functions [44, 45], and maladaptive [7]. However, when the individual is subjected to stress in early life, the stress response in adulthood may be shaped by prior experiences (Figure 1(b)). It was shown that depolarization-dependent release of glutamate in dorsal hippocampus is decreased in animals subjected to chronic stress in the prenatal life [117, 118]; however, little is known on the reprogramming of the stress response at the level of excitatory transmission induced by early-life stress. A very recent cross-sectional observational study examined the effects of early- and late-life trauma in Korean college students, showing a significant correlation between early trauma, stress, and psychological distress [184].

We speculate that the long-lasting attenuation of the stress response induced by early-life stress might also affect the changes in excitatory transmission usually induced by stress in adult life. Thus, hypothetically, both the rise of glutamate transmission induced by acute stress and the attenuation of excitatory currents caused by chronic stress might be affected by reprogramming of the stress response induced by early-life stress, thus likely leading to adaptive or maladaptive changes, depending on the intensities of the stressors. Experimental evidence is required to validate or falsify the hypothesis.

**Figure 2:**
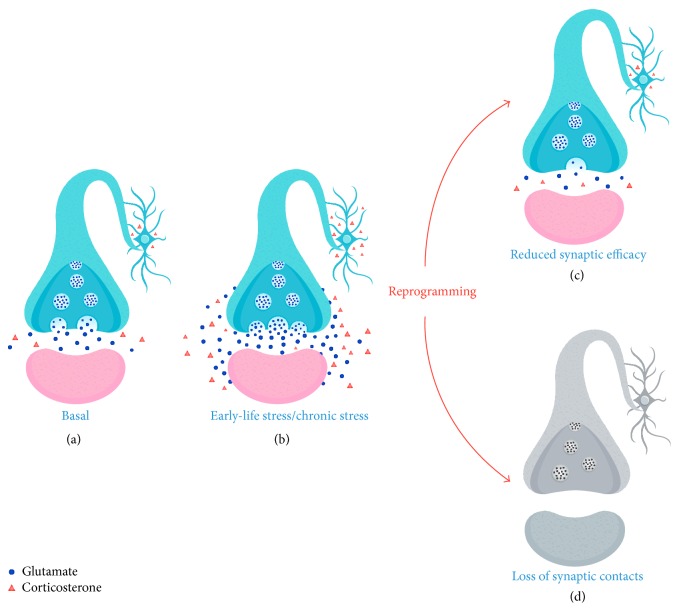
Long-term neuroplastic alterations induced by early-life stress and chronic stress. (a) Basal condition: presynaptic neuron (light blue), postsynaptic neuron (pink). (b) Repeated episodes of stress in early life or in adulthood induce an increase in glucocorticoids associated with a transient increase in glutamate release both in the synaptic cleft and in the extrasynaptic space. Increase in glutamate release may activate reprogramming mechanisms that lead to either reduced synaptic efficacy (c) or loss of synaptic contacts (d).

Excitotoxicity caused by excessive glutamate release and epigenetic reprogramming are reasonably among the main mechanisms involved in long-lasting neuroplastic alterations induced by stress [1, 185] (Figure 2). Excitoxicity is generally associated with reduced ability to clear the synaptic glutamate, resulting in glutamate spillover and activation of extrasynaptic glutamate receptors [32]. However, it is likely that exposure to high levels of corticosteroids (Figure 2(b)), together with inducing changes in excitatory transmission, activates epigenetic mechanisms, which modulate gene expression and neuronal responsiveness to stress. Of note, mounting evidence suggests that perinatal stress reprogramming of the neuroendocrine stress response and the ensuing behavioral state can cross multiple generations, thus supporting the hypothesis that epigenetic mechanisms underlie the reprogramming of the “stressed synapse” [22, 186]. This in turn leads to functional and structural consequences, in line with reduced synaptic efficacy [31, 117, 118] (Figure 2(c)) and number of synaptic contacts [52, 53, 105–108] (Figure 2(d)).

The mechanisms by which early-life events affect stress resilience via the reprogramming of the stress response and the modulation of excitatory neurotransmission warrant further investigation. In-depth studies of changes in glutamate transmission and dendrite remodeling induced by stress in early and late life will help to elucidate the biological underpinnings of the (mal)adaptive strategies the brain adopts to cope with environmental challenges in one's life.
